# Management of plant central metabolism by SnRK1 protein kinases

**DOI:** 10.1093/jxb/erac261

**Published:** 2022-06-16

**Authors:** Bruno Peixoto, Elena Baena-González

**Affiliations:** Instituto Gulbenkian de Ciência, Oeiras, Portugal and GREEN-IT Bioresources for Sustainability, ITQB NOVA, Oeiras, Portugal; Instituto Gulbenkian de Ciência, Oeiras, Portugal and GREEN-IT Bioresources for Sustainability, ITQB NOVA, Oeiras, Portugal; University College Cork, Ireland

**Keywords:** Carbon, central metabolism, homeostasis, nitrogen, SnRK1

## Abstract

SUCROSE NON-FERMENTING1 (SNF1)-RELATED KINASE 1 (SnRK1) is an evolutionarily conserved protein kinase with key roles in plant stress responses. SnRK1 is activated when energy levels decline during stress, reconfiguring metabolism and gene expression to favour catabolism over anabolism, and ultimately to restore energy balance and homeostasis. The capacity to efficiently redistribute resources is crucial to cope with adverse environmental conditions and, accordingly, genetic manipulations that increase SnRK1 activity are generally associated with enhanced tolerance to stress. In addition to its well-established function in stress responses, an increasing number of studies implicate SnRK1 in the homeostatic control of metabolism during the regular day–night cycle and in different organs and developmental stages. Here, we review how the genetic manipulation of SnRK1 alters central metabolism in several plant species and tissue types. We complement this with studies that provide mechanistic insight into how SnRK1 modulates metabolism, identifying changes in transcripts of metabolic components, altered enzyme activities, or direct regulation of enzymes or transcription factors by SnRK1 via phosphorylation. We identify patterns of response that centre on the maintenance of sucrose levels, in an analogous manner to the role described for its mammalian orthologue in the control of blood glucose homeostasis. Finally, we highlight several knowledge gaps and technical limitations that will have to be addressed in future research aiming to fully understand how SnRK1 modulates metabolism at the cellular and whole-plant levels.

## Teaser

Here we review studies linking the SnRK1 energy-sensing kinase and plant primary metabolism, and present a model in which SnRK1 function is centred on the maintenance of sucrose homeostasis.

## Introduction

All organisms need to adequately manage energy resources for optimal growth and survival. In plants, energy management occurs at the cellular and whole-organism levels through the coordination of metabolism, growth, and development in different organs (Smith and Stitt, 2007), affecting important traits such as stress resistance, branching, and seed filling. However, how the energy management network operates to coordinate carbon assimilation, storage, and growth is poorly understood.

One major component of the energy management network is SUCROSE NON-FERMENTING1 (SNF1)-RELATED KINASE 1 (SnRK1), a heterotrimeric Ser/Thr protein kinase complex harbouring a catalytic α-subunit and regulatory β- and γ- subunits. Early work identified SnRK1 as a key player in plant stress responses, being activated by stresses of different origin but that share a common low-energy denominator that is ultimately sensed by the SnRK1 kinase (Baena-González *et al.*, 2007; Baena-González and Sheen, 2008). Conversely, SnRK1 is inhibited by energy abundance in the form of various sugar phosphates, including trehalose 6-phosphate (Tre6P) (Toroser *et al.*, 2000; Zhang *et al.*, 2009; Piattoni *et al.*, 2011; Nunes *et al.*, 2013; Zhai *et al.*, 2018). Upon activation in response to low energy, SnRK1 implements an energy-conservation programme that promotes stress tolerance partly through inhibition of the target of rapamycin (TOR) kinase and thereby of energy-costly growth (Hulsmans *et al.*, 2016; Margalha *et al.*, 2019; Nukarinen *et al.*, 2016; Belda-Palazon *et al.*, 2020). Starvation-mediated activation of SnRK1 signalling also leads to deep metabolic readjustments, both through regulation of metabolic enzymes (Cho *et al.*, 2016; Nukarinen *et al.*, 2016) and through differential expression of >1000 genes (Baena-González *et al.*, 2007; Baena-González and Sheen, 2008; Pedrotti *et al.*, 2018; Wang *et al.*, 2021; Henninger *et al.*, 2022). Amongst these, many are related to anabolic and catabolic processes, and SnRK1 activation leads to their repression or induction, respectively. This transcriptional switch from anabolism to catabolism is mediated by the phosphorylation of S1- and C-class bZIP transcription factors (Ma *et al.*, 2011; Mair *et al.*, 2015; Pedrotti *et al.*, 2018; Henninger *et al.*, 2022), providing a more indirect way to affect metabolism in response to low carbon availability.

Besides its involvement in stress responses, an increasing number of reports attribute important functions to SnRK1 in the absence of external perturbations, implicating it in the daily maintenance of homeostasis and the coordination of metabolism and development (Radchuk *et al.*, 2010; Nukarinen *et al.*, 2016; Li *et al.*, 2020; Peixoto *et al.*, 2021; Liang *et al.*, 2021; Wang *et al.*, 2021; Henninger *et al.*, 2022). Molecular evidence for such basal activation of SnRK1 in the absence of stress was recently provided by the work of Jamsheer K *et al.* (2021) who showed that, under favourable conditions, TOR induces SnRK1 activity in an FCS-like zinc finger 8 (FLZ8)-dependent manner, as a way to limit its own activity. On the other hand, the transcriptional signature associated with the constitutive and moderate manipulation of SnRK1 is remarkably different from that associated with its strong activation by stress treatments or by transient overexpression of the catalytic subunit, suggesting that its functions under favourable conditions may differ from those under stress (Peixoto *et al.*, 2021).

The connections between SnRK1 and development have been recently reviewed (Baena-González and Hanson, 2017; Jamsheer K *et al.*, 2021). In this review, we therefore focus on the impact of SnRK1 on central metabolism, providing an overview of the enzyme and gene targets that have been described for SnRK1 in the main routes of carbon and nitrogen metabolism. We further review and discuss the metabolic alterations associated with the manipulation of SnRK1, focusing on the central and best understood catalytic α-subunit and on studies where no perturbations or stress treatments were applied.

## Carbon metabolism—sucrose

The first functional studies on SnRK1 were inspired by the ability of the plant kinase to complement the *snf1* mutant (Alderson *et al.*, 1991; Muranaka *et al.*, 1994) and were therefore focused on the possible transcriptional regulation of carbon metabolism by SnRK1. One early study revealed that silencing of the *PKIN1* gene, encoding one SnRK1α isoform in potato, caused reduced expression of the sucrose synthase (SUSY) gene *SUS4* and a >65% reduction in SUSY activity in tubers (Purcell *et al.*, 1998). Conversely, *PKIN1* overexpression led to an increase in *SUS4* transcript levels and 20–60% higher SUSY activities (McKibbin *et al.*, 2006). The connection between SnRK1 activity and SUSY expression and activity has thereafter been validated by many other studies characterizing tissue-specific or ubiquitous *SnRK1α* overexpression or silencing (Tiessen *et al.*, 2003; Radchuk *et al.*, 2010; Wang *et al.*, 2012; Jiang *et al.*, 2013; Wang *et al.*, 2017; Ren *et al*., 2018, 2019; Luo *et al.*, 2020; Liang *et al.*, 2021). SUSY plays a central role in Suc metabolism, in particular in sink tissues where, in the presence of UDP, it cleaves Suc into Fru and UDP-glucose (UDPGlc), whilst in the presence of ADP, it forms Fru and ADP-glucose (ADPGlc) (Stein and Granot, 2019). UDPGlc and ADPGlc are important precursors of cell wall and starch synthesis, respectively; by promoting the conversion of Suc into complex carbohydrates, SUSY promotes Suc influx into the sink and is therefore generally considered a marker of ‘sink strength’ (Zrenner *et al.*, 1995; Bieniawska *et al.*, 2007). Its regulation by SnRK1 suggests that SnRK1 may enhance sink capacity by promoting Suc consumption in sink tissues.

Suc can also be degraded by invertases (INVs), producing in this case Glc and Fru. *In vitro* assays with the potato vacuolar invertase StvacINV1 and its inhibitor protein StInvInh2B revealed that INV activity is controlled by an intriguing interplay between SnRK1β and SnRK1α subunits (Lin *et al.*, 2015): SnRK1β was able to suppress the inhibitory effect of StInvInh2B on StvacINV1, but pre-activated SbSnRK1 (also referred as StubSNF1) counteracted the effect of SnRK1β, thereby restoring StvacINV1 inhibition. A repression of INV activity by SnRK1 was further validated in cold-stored tubers, where SbSnRK1 overexpression caused an 83–95% reduction in INV activity whilst RNAi caused a 30–100% increase (Lin *et al.*, 2015). Interestingly, unlike lines overexpressing PKIN1 (Purcell *et al.*, 1998), SbSnRK1 overexpressors showed no alterations in SUSY activities (Lin *et al.*, 2015), indicating that the PKIN1 and SbSnRK1 α-subunits play different roles in carbon metabolism. In agreement with the work of Lin and colleagues, a more recent study in strawberry plants reported that FaSnRK1α overexpression inhibited both acid (AI) and neutral INV (NI) activities as well as the expression of the *FaNI* INV gene (Luo *et al.*, 2020). In potato, INV is the predominant enzyme for Suc cleavage during the active growth phase of stolons but, when tuberization is initiated, AI is replaced by SUSY to support the conversion of Suc into starch for storage (Viola *et al.*, 2001). Therefore, the induction of SUSY and the repression of INV activities by SnRK1 may reflect a positive effect of SnRK1 on the flux of Suc into starch, and a negative effect on the flux towards the glycolytic pathway and respiration. This interpretation is consistent with the observed phenotypes discussed in the next sections.

Early work employing protein extracts from various plant species as well as recombinant proteins showed that SnRK1 phosphorylates key enzymes of Suc biosynthesis (SUCROSE PHOSPHATE SYNTHASE, SPS), trehalose metabolism (TREHALOSE 6-PHOSPHATE SYNTHASE, TPS), carbon partitioning (FRUCTOSE-6-PHOSPHATE 2-KINASE/FRUCTOSE-2,6-BISPHOSPHATASE, F2KP), nitrogen assimilation (NITRATE REDUCTASE, NR), and isoprenoid biosynthesis (3-HYDROXY-3-METHYLGLUTARYL COA REDUCTASE, HMGR), resulting in all cases in the inactivation and/or recruitment of 14-3-3 proteins (McMichael *et al.*, 1995; Dale *et al.*, 1995; Ball *et al.*, 1995; Douglas *et al.*, 1997; Sugden *et al.*, 1999; Kulma *et al.*, 2004; Harthill *et al.*, 2006; Cho *et al.*, 2016; Robertlee *et al.*, 2017). *In vivo* evidence for the SnRK1-dependent phosphorylation of these enzymes has thereafter been obtained using phosphoproteomics analyses of gain- and loss-of-function SnRK1α mutants (Cho *et al.*, 2016; Nukarinen *et al.*, 2016) (see below).

SPS is an important enzyme in the Suc biosynthetic pathway, generating sucrose 6-phosphate (Suc6P) from fructose 6-phosphate (Fru6P) and UDPGlc, which can be further dephosphorylated by SUCROSE-PHOSPHATE PHOSPHATASE to Suc. In addition to the *in vitro* evidence that SnRK1 phosphorylates SPS (Sugden *et al.*, 1999), a relationship between elevated SnRK1 activity and reduced SPS activity has also been consistently observed *in vivo* in different tissues and species (Wang *et al.*, 2012; Jiang *et al.*, 2013; Wang *et al.*, 2017; Luo *et al.*, 2020). In some cases, lower SPS activity is accompanied by lower expression of *SPS* genes (Wang *et al.*, 2017; Luo *et al.*, 2020), suggesting a multilevel inhibitory effect over this metabolic enzyme by SnRK1. An impact on genes related to Suc metabolism, however, was not observed in Arabidopsis rosettes of SnRK1 gain- and loss-of-function mutants harvested at the end of the night or the end of the day (Peixoto *et al.*, 2021). Given the known diurnal changes in the expression of genes involved in central metabolism (Gibon *et al.*, 2004), it is possible that differences amongst genotypes were not detected due to the sample harvesting time.

The inhibition of SPS by SnRK1 would be expected to result in reduced Suc accumulation when SnRK1 is activated. However, expression of SnRK1α from a strong ubiquitous promoter (e.g. *35S*) consistently leads to increased Suc accumulation in leaves, fruits, and seeds of several plant species (Wang *et al.*, 2012; Jiang *et al.*, 2013; Wang *et al.*, 2017; Ren *et al.*, 2018; Wang *et al.*, 2019; Luo *et al.*, 2020; Liang *et al.*, 2021; Peixoto *et al.*, 2021). Overexpression of SnRK1α also leads to faster fruit ripening in tomato and strawberry, in agreement with the increased Suc content (Wang *et al.*, 2012; Yu *et al.*, 2018; Luo *et al.*, 2020) and SUSY activity (Luo *et al.*, 2020) of these plants.

How can the impact of SnRK1 on SPS activity be reconciled with the higher Suc content of SnRK1-overexpressing plants? A possible explanation could be that Suc accumulates due to decreased consumption rather than increased synthesis. However, SnRK1α overexpression was reported to lead to enhanced photosynthetic rates (Wang *et al.*, 2012; Jiang *et al.*, 2013; Liang *et al.*, 2021), and increased biomass accumulation (Wang *et al.*, 2012; Ren *et al.*, 2018; Liang *et al.*, 2021), arguing against this interpretation. Alternatively, SnRK1α overexpression may cause changes in the *in vivo* concentrations of SPS substrates (UDPGlc and Fru6P) and allosteric regulators (Glc6P as an activator and Pi as an inhibitor; Doehlert and Huber (1983) that override the inhibitory impact of phosphorylation by SnRK1 (at Ser158) (Winter and Huber, 2000). SPS activity measurements report the activation state of the enzyme, which reflects its phosphorylation status, and are performed in two different conditions of substrates, rate-limiting and nearly saturating concentrations. Therefore, the influence of *in vivo* concentrations of substrates and allosteric inhibitors is missing in these measurements. A moderate increase in UDPGlc, Fru6P, and Glc6P levels was indeed observed in SnRK1α1 overexpressor rosettes at Zeitgeber time (ZT) 4 and ZT8, with higher levels of these metabolites being accompanied by higher Suc levels at ZT8 when compared with ZT4 (Peixoto *et al.*, 2021). On the other hand, higher photosynthetic rates and higher concentrations of UDPGlc, Fru6P, and Glc6P may, at least partly, be explained by changes in F2KP activity. F2KP is a bifunctional enzyme whose product, fructose-2,6-bisphosphate (Fru-2,6bP), regulates carbon partitioning by controlling the interconversion between fructose-1,6-bisphophate (Fru-1,6-P2) and fructose-6-phosphate (Fru6P) (Nielsen *et al.*, 2004). By repressing the formation of Fru6P through cytosolic fructose-1,6-bisphosphatase (cyt-FBPase), Fru-2,6bP inhibits the synthesis of Suc, promoting the flux of carbon towards glycolysis. Although the functional outcome of F2KP phosphorylation by SnRK1 remains unclear, several lines of evidence suggest that SnRK1 activation may reduce Fru-2,6bP accumulation (Kulma *et al.*, 2004). First, in Arabidopsis leaves, Fru-2,6bP accumulation increased during the light period, peaking at the end of the day (Kulma *et al.*, 2004) when SnRK1 activity appears to be lowest (Peixoto *et al.*, 2021). Second, transfer of Arabidopsis cell cultures to fresh medium triggers Fru-2,6bP accumulation, and this can be blocked by the addition of the non-metabolizable Glc analogue 2-deoxyglucose, which activates AMPK in mammalian cells and SnRK1 in Arabidopsis cell cultures (Kulma *et al.*, 2004; Harthill *et al.*, 2006). In this context, regulation of F2KP activity by SnRK1 may lead to lower Fru-2,6bP levels and higher Fru6P and Suc accumulation which is consistent with the elevated Suc levels of SnRK1 overexpressors. Collectively, this suggests that, in the absence of perturbations, SnRK1 promotes Suc production and growth and that this may at least partly be accomplished by increasing photosynthetic capacity.

## Carbon metabolism—starch

Possible molecular connections to starch metabolism were evidenced by early studies on SnRK1. Silencing *SnRK1α* in wheat embryos led to reduced expression of *α-AMYLASE 2* (*α-AMY2*) expression (Laurie *et al.*, 2003), and similar findings were later on reported in rice (Lu *et al.*, 2007). Furthermore, seeds of the *snrk1a* rice mutant displayed severely retarded germination and early seedling growth, indicating that SnRK1 is important for the mobilization of starch reserves from the seed, at least partly by controlling the expression of *AMY* genes (Lu *et al.*, 2007).

Interestingly, SnRK1α2 was shown to interact with STARCH EXCESS 4 (SEX4) in a yeast two-hybrid assay (Fordham-Skelton *et al.*, 2002). However, whether this interaction occurs *in planta* remains to be assessed. SEX4 is involved in glucan dephosphorylation at the starch granule surface, a modification that is required for β-amylases and isoamylases to complete their hydrolytic functions (Streb and Zeeman, 2012). Interestingly, laforin, the functional equivalent of SEX4 in humans (Gentry *et al.*, 2007; Kötting *et al*., 2009), is phosphorylated by AMPK in a process that is essential for proper glycogen metabolism (Solaz-Fuster *et al.*, 2008; Romá-Mateo *et al.*, 2011). Nevertheless, whether SnRK1α2 and SEX4 interact *in planta* and whether this relates to the ability of SnRK1 to control starch degradation is unknown. Although two studies suggest that SnRK1 subunits, including the catalytic α-subunits, could localize to chloroplasts and starch granules (Fragoso *et al.*, 2009; Ruiz-Gayosso *et al.*, 2018), there is significant controversy regarding the binding of SnRK1 subunits to starch (Ávila-Castañeda *et al.*, 2014; Emanuelle *et al.*, 2015; Ruiz-Gayosso *et al.*, 2018).

A connection between SnRK1 and starch synthesis was also reported at the level of ADPGlc synthesis by ADPGlc pyrophosphorylase (AGPase). ADPGlc is the substrate for starch biosynthesis in higher plants and its production is the first committed step of starch biosynthesis. Several studies have reported increased AGPase activity in leaves, tubers, and storage roots of plants overexpressing SnRK1α [PKIN1 in the case of potato; McKibbin *et al.*, 2006; Wang *et al.*, 2012; Jiang *et al.*, 2013; Wang *et al.*, 2017; Ren *et al.*, 2018; Liang *et al.*, 2021). Such an increase in AGPase activity could be due to the increased *AGPase* gene expression reported in some of the studies (McKibbin *et al.*, 2006; Wang *et al.*, 2017) or could be related to the redox activation of AGPase or some other mechanism. AGPase is redox regulated by light and metabolites, requiring reduction of a cysteine residue in the enzyme for its monomerization and activity (Streb and Zeeman, 2012). Consistent with such metabolite-dependent control, the redox activation state of AGPase declines within 2 h in excised potato tuber discs if sugars are not provided exogenously (Tiessen *et al.*, 2002). In this system, silencing of *PKIN1* led to a more rapid inactivation of AGPase in sugar-deprived tuber discs, with delayed AGPase activation upon Suc supplementation (Tiessen *et al.*, 2003), altogether suggesting a positive role for SnRK1 in starch synthesis and highlighting its possible relevance for responding to alterations in Suc supply (e.g. during the day–night transitions) *in planta*. In addition to AGPase, increased STARCH SYNTHASE (SS) activity has been reported for plants overexpressing SnRK1α (Wang *et al.*, 2017; Ren *et al.*, 2019; Liang *et al.*, 2021), with *SS* genes shown to be up-regulated in two of the studies (Wang *et al.*, 2017; Ren *et al.*, 2019).

Collectively, these studies suggest that SnRK1 promotes starch degradation, partly by inducing the expression of *AMY* genes, and that it also promotes starch synthesis through the up-regulation of AGPase and SS activities. These two contrasting outcomes are likely to be associated with different developmental stages and tissues, with starch synthesis being promoted in leaves and Suc-importing growing sinks, and starch degradation being promoted when growth is dependent on the remobilization of stored energy (e.g. germinating seeds or tubers). Such a conclusion is supported by numerous studies performed in a wide range of species and tissues. In barley, antisense-mediated silencing of *SnRK1α* in pollen led to defects in starch accumulation and pollen abortion (Zhang *et al.*, 2002). In the moss *Physcomitrium patens*, full *SnRK1α* knock-out (*snf1a/snf1b*) resulted in defective starch accumulation, with plants requiring constant illumination or exogenous sugar supply to survive (Thelander *et al.*, 2004). A recent metabolic characterization of a SnRK1α1 overexpressor and a *snrk1α1*^*–/–*^*snrk1α2*^*+/–*^ knockdown line also showed a subtle but significant effect on starch accumulation, with the overexpressor accumulating more starch and the loss-of-function mutant accumulating less (Peixoto *et al.*, 2021). While the impact of SnRK1 on starch remobilization in germinating seeds and tubers is likely to be direct through regulation of genes and enzymes involved in starch degradation, the impact of SnRK1 on starch accumulation in leaves and Suc-importing sinks may be indirect. Given the intimate connection between Suc and starch metabolism (Streb and Zeeman, 2012) and the impact of SnRK1 on Suc accumulation, it is possible that the increased starch content of SnRK1α1 overexpressors is due to the increased Suc levels reported in these plants, with the opposite being the case for the SnRK1 loss-of-function mutants.

Adding further complexity, the starch phenotypes reported for SnRK1 mutants are in some cases conflicting, with Arabidopsis SnRK1α1 overexpressors accumulating lower starch levels than wild-type plants in response to sugar supplementation (Jossier *et al.*, 2009), in contrast to what is reported under normal growth conditions (McKibbin *et al.*, 2006; Wang *et al.*, 2012; Jiang *et al.*, 2013; Wang *et al.*, 2017; Ren *et al.*, 2018; Liang *et al.*, 2021). An excess starch phenotype was in turn reported for Arabidopsis plants where *SnRK1α* was strongly silenced via virus-induced gene silencing (Baena-González *et al.*, 2007), which contrasts with the defective starch accumulation reported for the *snrk1α* mutant in *P. patens* (Thelander *et al.*, 2004) and the Arabidopsis knockdown line (Peixoto *et al.*, 2021). A possible explanation for such controversy could be that in some cases (moderate changes in SnRK1 activity), a change in starch levels reflects direct regulation of the starch synthesis or degradation pathways, whilst in others (severe changes in SnRK1 activity) additional indirect effects are at play due to changed growth and development.

## Carbon metabolism—Tre6P signaling

Class II TPS proteins were also amongst the first targets described for SnRK1 (Harthill *et al.*, 2006; Cho *et al.*, 2016; Nukarinen *et al.*, 2016), but the functional relevance of this phosphorylation is still unknown. Class II TPS proteins lack TPS catalytic activity and have been hypothesized to play regulatory roles (Ramon *et al.*, 2009; Delorge *et al.*, 2015), potentially in Tre6P metabolism and signalling. In Arabidopsis, Tre6P is synthesized from UDPGlc and Glc6P by TPS1, and it is dephosphorylated by trehalose 6-phosphate phosphatases (TPPs), yielding trehalose (Avonce *et al.*, 2006). Tre6P is a central signalling molecule that maintains Suc homeostasis by signalling Suc availability and acting as a feedback regulator of Suc synthesis and consumption, in what is often referred to as the Suc–Tre6P nexus (see Fichtner and Lunn, 2021 for details). Tre6P down-regulates Suc synthesis partly by diverting the flux of carbon into the synthesis of organic acids, and, during the night, by slowing down the rate of starch mobilization (Martins *et al.*, 2013; Figueroa *et al.*, 2016). On the other hand, the mechanisms by which an increase in Suc results in increased Tre6P levels appear to be unrelated to TPS1 accumulation (Yadav *et al.*, 2014) and thus far remain unknown.

Tre6P is also a known allosteric inhibitor of SnRK1α activity in the micromolar range, which is compatible with the concentrations found in both Arabidopsis rosettes and Suc-fed seedlings (Lunn *et al.*, 2006; Zhang *et al.*, 2009; Martins *et al.*, 2013; Nunes *et al.*, 2013). This inhibitory effect requires a proteinaceous factor that could, at least partly, correspond to the SnRK1-activating kinases (SnAKs), since Tre6P has been shown to disrupt the interaction of SnRK1 with SnAK, hence reducing SnRK1 phosphorylation and activity (Zhai *et al.*, 2018).

Despite the clear link between Tre6P and SnRK1, much less is known about the impact of SnRK1 on Tre6P metabolism. According to the Suc–Tre6P nexus model, SnRK1-mediated changes to Suc are expected to result in concomitant changes to Tre6P accumulation (Yadav *et al.*, 2014). Indeed, a clear correlation between Suc and Tre6P levels is also observed in SnRK1α gain- and loss-of-function mutants, as reported for Arabidopsis rosettes during the day–night cycle, and in pea embryos across the different stages of embryo development (Radchuk *et al.*, 2010; Peixoto *et al.*, 2021). However, the relationship between Suc and Tre6P appears to be altered when SnRK1 is manipulated. Compared with wild-type Arabidopsis plants, Tre6P:Suc ratios were up to 1.9-fold higher in the rosettes of a SnRK1α1 overexpressor line and 2.8-fold lower in those of the *sesquiα2* loss-of-function mutant (Peixoto *et al.*, 2021). These differences were mostly due to changes in Tre6P levels, with Tre6P hyperaccumulating in response to Suc in the SnRK1α1 overexpressor and hypoaccumulating in the loss-of-function mutant. In addition, the differences were not constant, but increased markedly when Suc levels peaked at the end of the day, altogether suggesting that SnRK1 is part of the mechanism that links Suc to Tre6P. The relationship between Suc and Tre6P has been explored in great detail over a wide range of growth conditions, tissues, and plant species, and high Tre6P:Suc ratios have been typically found associated with metabolically active tissues (Lunn *et al.*, 2014). Given the association between SnRK1 and growth repression, it was unexpected that overexpression of SnRK1α1 also led to higher Tre6P:Suc ratios. However, this observation is in accordance with the view that SnRK1 is also required for growth under favourable conditions (Margalha *et al.*, 2019; Baena-González and Lunn, 2020). A positive effect of SnRK1 on Tre6P accumulation is nevertheless not always observed. In Arabidopsis plants overexpressing peach SnRK1α1, Tre6P levels were mildly reduced compared with the wild type (Zhang *et al.*, 2021). However, Tre6P was quantified using ELISA-based immunodetection, resulting in values that were more than one order of magnitude higher than those obtained for Arabidopsis seedlings by LC/MS (Lunn *et al.*, 2014), suggesting a possible contribution of other sugars to the obtained values. On the other hand, silencing *SnRK1α* in pea embryos led to elevated Tre6P levels that matched the high accumulation of Suc, probably as a result of impaired embryo growth (Radchuk *et al.*, 2010).

It is not yet known how the Suc status is perceived at the molecular level, or how this information is then conveyed to regulate Tre6P levels, but such a pathway may involve altered Tre6P synthesis by TPS1, altered Tre6P dephosphorylation by TPP enzymes, or both. However, the altered levels of Tre6P detected in the SnRK1α mutant lines could not be fully explained either by changes in TPS1 protein abundance or by the transcriptional behaviour of the *TPS/TPP* genes (Peixoto *et al.*, 2021). Since the currently available data on transcript and protein abundance fail to explain the observed Tre6P phenotypes, it is possible that post-translational mechanisms affecting enzyme activities might be at play to control how much Tre6P accumulates.

Regardless of the underlying mechanism, the relationship between Suc and Tre6P appears to be altered when SnRK1 is manipulated, suggesting that, besides directly regulating Suc metabolism, SnRK1 could be involved in the sensitization to Suc signals.

## Lipid metabolism

Triacylglycerol (TAG) is the most important form of seed storage oil (Ohlrogge and Chapman, 2011; Chapman and Ohlrogge, 2012). TAG accumulates in the form of oil bodies, and its breakdown into fatty acids (FAs) and glycerol (Graham, 2008) can be used by the germinating seedling to fuel heterotrophic growth and gluconeogenesis (Baker *et al.*, 2006). A connection between SnRK1 and lipid metabolism came from the identification of a putative SnRK1 target motif in the sequence of DIACYLGLYCEROL TRANSFERASE 1 (DGAT1) from *Tropaeolum majus* (garden nasturtium). DGAT1 plays an important regulatory role during TAG assembly by controlling how much carbon flows into TAG synthesis (Weselake *et al.*, 2008). Mutagenesis of Ser197 to Ala in *T. majus* DGAT1 resulted in a 38–80% increase in recombinant DGAT1 activity. Overexpression of the DGAT^Ser197A^ phosphomutant variant in Arabidopsis also led to a 20–50% increase in oil content on a per seed basis (Xu *et al.*, 2008). More recent work with recombinant, lipidated DGAT1 from *Brassica napus* (rapeseed) showed this enzyme to be phosphorylated by SnRK1 *in vitro*, losing up to 40% of its catalytic activity after 30 min of incubation with the kinase (Caldo *et al.*, 2018).

Branching off from the TAG biosynthetic pathway, and parallel to the DGAT1 reaction, *de novo* phosphatidylcholine (PC) biosynthesis can also occur, competing for the available diacylglycerol (DAG) pool (Nakamura, 2021). Besides being a major constituent of the plasma membrane, PC is a precursor for the synthesis of free polyunsaturated FAs in the endoplasmic reticulum (Browse and Somerville, 1991) and glycerolipids in the plastid membranes (Ohlrogge and Browse, 1995), and serves as a reservoir for secondary messenger molecules (Exton, 1990). SnRK1 was recently found to phosphorylate one of the enzymes involved in PC biosynthesis, CTP:PHOSPHOCHOLINE CYTIDYLYLTRANSFERASE (CCT), which is responsible for transferring a cytidyl moiety from CTP to phosphocholine, yielding CDP-choline (Inatsugi *et al.*, 2002). Phosphorylation of Arabidopsis CCT1 by SnRK1 led to 70% inhibition of its enzymatic activity (Caldo *et al.*, 2019). Furthermore, transient co-expression of SnRK1 with CCT1 in *Nicotiana benthamiana* leaves blocked the PC accumulation induced by CCT1 in this system, providing also *in vivo* evidence for the SnRK1-mediated inhibition of CCT1 (Caldo *et al.*, 2019). In plants, storage lipids such as TAG are mainly stored as seed oil bodies which are surrounded by a monolayer of PC molecules (Huang, 2018). Due to this tight relationship between TAG and PC, Caldo and colleagues hypothesized that co-regulation of CCT1 and DGAT1 by SnRK1 could be important for synchronizing the two metabolic pathways, enabling a reduction in oil body formation when carbon is limiting (Caldo *et al.*, 2019).

Besides direct enzyme regulation, SnRK1 also regulates lipid synthesis at the transcriptional level by interacting with transcription factors. One such interactor is WRINKLED1 (WRI1), which controls the expression of genes involved in the late steps of glycolysis and plastidial lipid biosynthesis in Arabidopsis (Ruuska *et al.*, 2002; Baud *et al.*, 2007; Maeo *et al.*, 2009). Mutations in *WRI1* led to an ~80% reduction in FA and TAG content and a marked decrease in the flux of carbon from sugar to pyruvate, at the level of plastidial glycolysis (Focks and Benning, 1998). Recent work has demonstrated that SnRK1 phosphorylates WRI1 on Thr70 and Ser166, targeting it for proteasomal degradation (Zhai *et al.*, 2017). A functional connection between SnRK1 and WRI1 is further supported by the finding that overexpression of SnRK1 blocks WRI1-mediated FA biosynthesis and TAG accumulation in *N. benthamiana* leaves (Zhai *et al.*, 2017).

Another important transcription factor upstream of WRI1 is FUSCA3 (FUS3). FUS3 plays important roles during seed development, promoting both seed dormancy and oil accumulation (Keith *et al.*, 1994; Meinke *et al.*, 1994; Tiedemann *et al.*, 2008; Roscoe *et al.*, 2015). Overexpression of FUS3 results in increased oil accumulation in young Arabidopsis seedlings and Bright Yellow 2 (BY2) cell cultures (Zhang *et al.*, 2016). Although *WRI1* was up-regulated by FUS3 overexpression in Arabidopsis seedlings, this was not accompanied by an induction of well-established WRI1 target genes, suggesting that the positive effect of FUS3 on lipid biosynthesis is at least partially independent of WRI1 (Zhang *et al.*, 2016). FUS3 is also phosphorylated by SnRK1, but, in this case, phosphorylation of its N-terminus leads to enhanced FUS3 protein stability and accumulation (Tsai and Gazzarrini, 2012). Although the stabilization of FUS3 could suggest a positive effect of SnRK1 on lipid biosynthesis, the fact that Arabidopsis seeds overexpressing SnRK1α have reduced oil content (Zhai *et al.*, 2017) and the fact that SnRK1 inhibits TAG synthesis and oil deposition by other mechanisms (Weselake *et al.*, 2008; Zhai *et al.*, 2017; Caldo *et al.*, 2018) argue against this hypothesis. The connection between SnRK1 and FUS3 could hence relate to other functions of the transcription factor, such as in promoting seed dormancy.

In addition to the effect on lipid synthesis, SnRK1 has been implicated in the mobilization of TAG reserves during seed germination, with an inducible loss-of-function mutant showing persistently elevated TAG levels during the first 7 d (Henninger *et al.*, 2022). The inability of the *snrk1α* mutant to readily mobilize lipid reserves was accompanied by a down-regulation of *SUGAR DEPENDENT 1* (*SDP1*), encoding one of the main lipases involved in FA release from TAG stores during seed germination (Eastmond, 2006; Quettier and Eastmond, 2009), as well as by the down-regulation of *ACYL-COA OXIDASE 4* (*ACX4*) and *PEROXISOMAL MALATE DEHYDROGENASE 2* (*PMDH2*), encoding two enzymes involved in FA β-oxidation (Adham *et al.*, 2005; Pracharoenwattana *et al*., 2007, 2010). Nevertheless, these transcriptional changes were very mild and only affected a few genes, altogether suggesting that SnRK1 controls TAG catabolism mostly post-transcriptionally. It is also possible, as proposed by the authors of the study, that SnRK1-dependent transcriptional control is centred around just a few critical genes encoding rate-limiting enzymes of these pathways (Henninger *et al.*, 2022).

Altogether, these studies reveal that SnRK1 regulates lipid metabolism at the transcriptional and post-transcriptional levels, restricting lipid synthesis by inhibiting FA, TAG, and PC production, but also promoting TAG breakdown. The net result of SnRK1 action would be an efficient mobilization of lipid reserves during germination and seedling establishment. On the other hand, an inhibitory effect of SnRK1 on lipid accumulation is hard to reconcile with studies showing that SnRK1 is necessary for proper seed filling in pea (Radchuk *et al*., 2006, 2010). It is possible that a local and transient effect of SnRK1 on storage lipid synthesis during the seed-filling stage is masked by additional effects derived from the severity of *SnRK1α* silencing.

## Glycolysis and the TCA cycle

The conversion of Glc into pyruvate through glycolysis serves as a bridge between carbon metabolism and the tricarboxylic acid (TCA) cycle. Besides producing ATP and reducing power, glycolysis and the TCA cycle provide carbon skeletons for the synthesis of amino acids, forming one of the bases of macromolecule synthesis.

SnRK1 has been implicated in the regulation of several glycolysis-related enzymes, including pyruvate kinase (PK) (Beczner *et al.*, 2010). PK interacts in yeast two-hybrid assay with the two SnRK1 catalytic subunits of potato (PKIN1 and StubSNF1). In addition, the corresponding antisense potato lines show defects in the daily patterns of leaf PK activities, suggesting that SnRK1 is required for the proper timing and extent of PK activation (Beczner *et al.*, 2010). These defects could not be correlated to changes in the expression of the *PKc* gene, implicating post-transcriptional mechanisms in PK control. Nevertheless, it remains unclear from this study whether SnRK1 indeed phosphorylates PK and, if so, what is the functional outcome of this phosphorylation.

SnRK1 was also shown to phosphorylate the non-phosphorylating glyceraldehyde-3-phosphate dehydrogenase (np-Ga3PDHase) (Piattoni *et al.*, 2011), leading to its interaction with 14-3-3 and subsequent inactivation (Bustos and Iglesias, 2003). np-Ga3PDHase oxidizes glyceraldehyde-3-phosphate (Ga3P) to 3-phosphoglycerate (3-PGA), generating NADPH instead of NADH and ATP. By phosphorylating and inactivating np-Ga3PDHase, SnRK1 was proposed to limit NADPH and pyruvate production, thereby favouring the flux of carbon towards starch synthesis over reductive biosynthetic processes and respiration (Piattoni *et al.*, 2011). It is important to note that such regulation was only observed in the endosperm and shoot tissues, and not in leaves, showing its specificity for storage tissues.

SnRK1 may also influence glycolytic rates by targeting F2KP. Besides a repressive effect on Fru6P formation and thereby on Suc synthesis (see section on Suc metabolism), Fru-2,6bP also promotes the activity of pyrophosphate:fructose 6-phosphate phosphotransferase (PFP) (Nielsen *et al.*, 2004). PFP is a unique component of plant glycolysis that catalyses the PPi-dependent formation of Fru-1,6-P2, hence bypassing the ATP-dependent reaction catalysed by phosphofructokinase. PFP allows increased flexibility and flux in plant glycolysis, especially in conditions where Fru-2,6bP increases, such as in metabolically active and growing tissues (Stitt, 1990; Stitt and Sonnewald, 1995). Nevertheless, it remains to be clarified if F2KP phosphorylation by SnRK1 in this context promotes or down-regulates glycolysis.

Only a few studies have actually quantified the levels of glycolytic intermediates in SnRK1α mutants. Rosettes of Arabidopsis SnRK1α overexpressors show no significant changes in the levels of glycolytic intermediates (Peixoto *et al.*, 2021), while rosettes of loss-of-function mutants have reduced levels of 3-phosphoglycolate (3-PGA), phospho*enol*pyruvate (PEP), and pyruvate (Nukarinen *et al.*, 2016; Peixoto *et al.*, 2021). However, silencing *SnRK1α* specifically in potato tubers results in unchanged pyruvate and PEP levels (Tiessen *et al.*, 2003), whilst silencing it in pea embryos leads to increased 3-PGA levels (Radchuk *et al.*, 2010). The reason behind these conflicting reports is unclear. On the one hand, the reduced accumulation of glycolytic intermediates in *snrk1α* knockdown rosettes is consistent with the defects in PK activities observed in SnRK1 loss-of-function tubers and the established role of animal AMPK as a positive regulator of glycolysis (Herzig and Shaw, 2018). On the other hand, the fact that SnRK1 manipulation does not consistently alter the levels of glycolytic intermediates may be explained by the centrality of the glycolytic pathway. Being at the core of metabolism, glycolysis is likely to be buffered by a constant exchange of intermediates with sister pathways, thus making it difficult to detect defects using static metabolite analyses.

Reduced levels of glycolytic intermediates could also result, for example, from increased carbon flow towards downstream metabolic pathways, such as the TCA cycle. The TCA cycle is involved in the synthesis of organic acids, that serve as carbon skeletons for nitrogen assimilation and amino acid synthesis. In animal cells, AMPK promotes the flux of carbon into the TCA cycle by maintaining pyruvate dehydrogenase (PDH) activity (Cai *et al.*, 2020) and thereby acetyl-CoA production from pyruvate (Sun *et al.*, 2015).

In plants, thus far, there are no reports linking SnRK1 to particular enzyme(s) of the TCA cycle. However, a few studies have reported changes in organic acid levels when SnRK1 is genetically manipulated. In Arabidopsis *snrk1α* knockdown rosettes, several organic acids do accumulate to a higher extent than in the wild type, consistent with the idea that SnRK1 may inhibit the flow of carbon into the TCA cycle (Nukarinen *et al.*, 2016; Peixoto *et al.*, 2021). However, a mild increase in, for example, fumarate and 2-oxoglutarate (2-OG) levels, was also reported in the leaves of SnRK1α1 overexpressors (Liang *et al.*, 2021; Peixoto *et al.*, 2021). On the other hand, in pea embryos, the only example of sink tissues where organic acids have been measured, silencing SnRK1 leads to higher levels of 2-OG and lower levels of malate and fumarate (Radchuk *et al.*, 2010). Although this could be taken as evidence that SnRK1 promotes the usage of carbon skeletons for amino acid synthesis, it is more likely that the accumulation of 2-OG is caused by the inability of the embryo to grow when SnRK1 is depleted. The interpretation of metabolite data related to the TCA cycle is particularly challenging in plants, where numerous anaplerotic routes exist to replenish the cycle at different stages (Sweetlove *et al.*, 2010) and where intermediates are used for many processes beyond ATP production and amino acid synthesis, such as the production of root exudates (Badri and Vivanco, 2009; Sweetlove *et al.*, 2010). The accumulation of a particular metabolite could therefore be due to increased general flow through the cycle, increased incorporation through a particular anaplerotic route, or decreased utilization in downstream processes.

Nevertheless, the metabolic fingerprint of *snrk1α* knockdown mutants, where reduced accumulation of glycolytic intermediates is coupled to increased accumulation of several organic acids (Nukarinen *et al.*, 2016; Peixoto *et al.*, 2021), is reminiscent of the one obtained upon a transient increase in Tre6P levels (Figueroa *et al.*, 2016). Figueroa and colleagues showed that a transient increase in Tre6P results in the post-translational activation of PEP CARBOXYLASE (PEPC) and NR, altogether contributing to an increased diversion of photoassimilate towards the TCA cycle and amino acid synthesis (Figueroa *et al.*, 2016). It is therefore tempting to speculate that the effects of Tre6P on metabolism are via inhibition of the SnRK1 kinase. In the case of NR, the potentially increased diversion of organic acids towards nitrogen assimilation of the *snrk1α* knockdown mutants is consistent with the established connection between SnRK1 and NR (see below for more details). In the case of PEPC, the fact that this activatory phosphorylation on Ser11 was moderately reduced under steady-state condition in a SnRK1α overexpresor (Nukarinen *et al.*, 2016) is also consistent with such a hypothesis.

## Nitrogen and amino acid metabolism

Soil inorganic nitrogen is the main form of plant-available nitrogen, and in most soils is present as nitrate (NO^3–^) (Harmsen and Kolenbrander, 1965). Nitrate is taken up at the root level via plasma membrane-localized nitrate transporters and is reduced to nitrite (NO^2–^) by the cytoplasmatic enzyme NR. NR was one of the very first SnRK1 targets to be identified using *in vitro* assays and, similarly to most other SnRK1 enzyme targets described so far, it was shown to be targeted and inactivated by 14-3-3 proteins after phosphorylation (McMichael *et al.*, 1995; Moorhead *et al.*, 1996; Sugden *et al.*, 1999). *In vivo* evidence for the phosphorylation of NR by SnRK1 was later obtained from phosphoproteomic analyses of Arabidopsis SnRK1α overexpressors and knockdown mutants (Nukarinen *et al.*, 2016). The inhibitory function of SnRK1 over NR was also substantiated *in planta*, with SnRK1α overexpressor lines showing reduced enzyme activity under control conditions (Wang *et al.*, 2012) and in response to sugar supplementation (Jossier *et al.*, 2009). Reduced NR activity impacts on nitrogen metabolism, resulting in decreased levels of soluble protein in SnRK1α overexpressors (Wang *et al.*, 2012; Yu *et al.*, 2018) and in moderately increased levels (1.6-fold) in a *snrk1α* knockdown mutant (Peixoto *et al.*, 2021). A negative impact of SnRK1 on nitrogen assimilation is further supported by a recent study on the diel regulation of storage protein synthesis in maize (Li *et al.*, 2020). The OPAQUE2 (O2) transcription factor controls genes encoding zein, the main nitrogen storage compound of maize seeds (Mertz *et al.*, 1964). Li and colleagues showed that SnRK1 may indirectly repress O2 activity by destabilizing a protein that promotes O2 nuclear translocation. This mechanism allows not only the limitation of zein synthesis in response to sudden carbon shortage, but also the adjustment of zein synthesis to the daily Suc fluctuations (Li *et al.*, 2020). Although this study did not use SnRK1α gain- or loss-of-function mutants to measure storage protein levels, such a mechanism would be expected to result in enhanced storage protein accumulation when SnRK1 is depleted. A more general impact of SnRK1 in protein synthesis is also suggested by a connection with TOR, a positive regulator of translation (Sesma *et al.*, 2017). SnRK1 interacts with TOR (Nukarinen *et al.*, 2016; Belda-Palazón *et al.*, 2020) and represses it in response to abscisic acid and energy deprivation (Belda-Palazón *et al.*, 2020). Furthermore, numerous components of the translation machinery were hyperphosphorylated in the rosettes of a *snrk1α* knockdown mutant (Nukarinen *et al.*, 2016), suggesting that SnRK1 inhibits their phosphorylation directly or indirectly.

SnRK1 mutations also have an impact on the levels of several amino acids. A 2-fold increase in Glu levels was reported for a tomato SnRK1α overexpressor, potentially explaining the up-regulation of the *GLUTAMATE DEHYDROGENASE 2* (*GDH2*) gene observed in the same line (Liang *et al.*, 2021). Conversely, an Arabidopsis inducible loss-of-function mutant had a 25% reduction in Glu levels and this was accompanied by a 21% increase in the Gln content (Nukarinen *et al.*, 2016). A change in the Glu/Gln ratio could be explained by an increased flow of nitrogen via NR when SnRK1 is depleted, leading to higher rates of Glu to Gln conversion by GLUTAMINE SYNTHASE (GS).

Asn is also enriched in the source tissues of *snrk1α* knockdown mutants (Nukarinen *et al.*, 2016). Given the major role of Gln and Asn in the transport of nitrogen to sink tissues (The *et al.*, 2021), their enrichment in the source leaves of the *snrk1α* mutant may indicate, besides increased synthesis, also decreased export. Overall, and as seen for other metabolites discussed in previous sections, the effect of constitutive and moderate SnRK1α depletion in Arabidopsis (Nukarinen *et al.*, 2016; Peixoto *et al.*, 2021) contrasted strongly with that of severe *SnRK1α* silencing in pea embryos (Radchuk *et al*., 2006, 2010) The latter resulted in an 18% and 16% reduction in the levels of globulin and albumin, respectively, the main storage proteins of legumes (Radchuk *et al.*, 2010), and reduced accumulation of both Gln and Asn (Radchuk *et al.*, 2010). Whether these conflicting outcomes relate to the severity of SnRK1 manipulation in the latter, or to some other process, remains to be assessed. It is nevertheless tempting to speculate that the Gln/Asn enrichment reported in rosettes and the Gln/Asn depletion reported in embryos could relate to defects in the coordination of nitrogen metabolism between source and sink tissues, or that proper SnRK1 signalling is required for sink tissues to import sufficient nitrogen compounds for fuelling protein synthesis. Strikingly, SnRK1 appears also to be required for amino acid synthesis: silencing *SnRK1α* during germination and early seedling development led to markedly reduced levels of most amino acids, including Asn and Gln, that could not be fully explained by defects in protein degradation (Henninger *et al.*, 2022). Furthermore, this was accompanied by a lower expression of genes involved in amino acid synthesis, in particular those of Ser, Gly, and Cys metabolism (Henninger *et al.*, 2022).

Lower levels of Ser and Gly were also reported for Arabidopsis rosettes and pea embryos depleted of SnRK1α (Radchuk *et al.*, 2010; Nukarinen *et al.*, 2016). Given that the biosynthetic pathway for Ser, Gly, and Cys branches out from the glycolytic pathway at the level of 3-PGA, reduced accumulation of Ser and Gly could be partly explained by the lower 3-PGA levels of the *snrk1α* knockdown mutant (Peixoto *et al.*, 2021; see previous section). More surprising is the accumulation of Cys reported in one of these studies (Radchuk *et al.*, 2010). However, Cys can serve as a sink for sulfur, and a link between SnRK1 and sulfur metabolism was recently identified at the transcriptional level. The Arabidopsis *snrk1α* mutant shows a sulfur starvation transcriptional signature that includes the up-regulation of *SERINE ACETYLTRANSFERASE 3;2* (*SERAT3;2*) involved in Cys synthesis from Ser (Peixoto *et al.*, 2021).

Besides nitrogen assimilation and protein and amino acid synthesis, SnRK1 also regulates amino acid degradation. Silencing *SnRK1α* during seed germination led to defects in seedling establishment that could be partly attributed to defects in amino acid catabolism for fuelling growth (Henninger *et al.*, 2022). During the first days after germination, and prior to exposure to light, the *snrk1α* mutant showed significantly delayed degradation of storage proteins (e.g. globulins) and decreased accumulation of total amino acids. This could partly be explained by the decreased expression of many genes involved in amino acid catabolism, such as *BRANCHED CHAIN AMINO ACID TRANSAMINASE2* (*BCAT2*) or *METHYLCROTONYL-COA CARBOXYLASE SUBUNIT A/B* (*MCCA/B*), which were strongly down-regulated in the *snrk1α* knockdown mutant (Henninger *et al.*, 2022). Most interestingly, the down-regulation of these genes was accompanied by reduced expression of *PYRUVATE ORTHOPHOSPHATE DIKINASE* (*PPDK*). Pyruvate resulting from amino acid breakdown is used by PPDK to produce PEP, which in turn is used in gluconeogenesis to generate Glc for seedling growth (Eastmond *et al.*, 2015). *PPDK* expression was further shown to be regulated by the bZIP63 transcription factor, a direct target of SnRK1 (Mair *et al.*, 2015).

Collectively, these studies reveal important stress-independent functions of SnRK1 in nitrogen metabolism, down-regulating nitrogen assimilation, protein synthesis, and amino acid metabolism, and promoting the catabolism of proteins and amino acids.

## Concluding remarks

SnRK1 kinases are crucial for the adequate distribution of resources in situations of stress, thereby promoting homeostasis and stress tolerance. Nevertheless, their function is not restricted to stress responses, and mounting evidence implicates SnRK1 in the fine-tuning of metabolism and other processes also during normal growth and development. In this review, we have considered studies that: (i) mechanistically link SnRK1α to specific aspects of primary metabolism; (ii) characterize gain- and loss-of-function SnRK1α mutants in the context of primary metabolism; and (iii) do not apply stress treatments or rely on the acute overexpression of components of SnRK1 signalling, as this largely mimics the stress-triggered starvation response (Baena-González *et al.*, 2007; Baena-González and Sheen, 2008) that is likely to override other SnRK1 functions. We propose the following model for the homeostatic control of metabolism by SnRK1 kinases, in an attempt to collectively explain the effects observed on various aspects of metabolism in different organs and developmental stages (Supplementary Table S1).

**Fig. 1.**
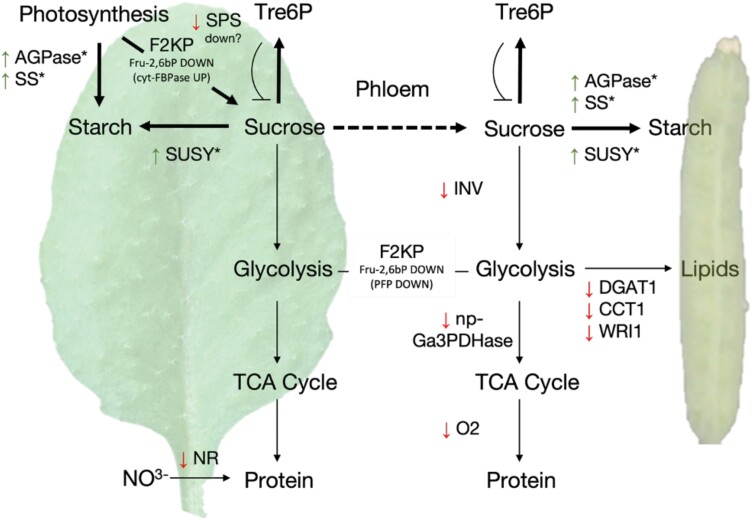
Model of SnRK1-mediated metabolic adaptations in source and actively growing sink tissues. SnRK1 acts by regulating metabolic enzymes and transcription factors (see text for details), leading to an increase (green arrows) or decrease (red arrows) in their activities. Lack of green/red arrows in F2KP indicates that the outcome of SnRK1-mediated regulation on enzyme activity is unclear. In source and actively growing sink tissues, SnRK1 enhances carbon input, either by increasing photosynthetic rates or by promoting carbon import from the phloem. Carbon is further directed towards the synthesis of starch and sucrose. The negative impact of SnRK1-mediated phosphorylation on SPS may be overriden by changes in the accumulation of SPS allosteric regulators, resulting in high sucrose synthesis (see text for details). This mechanism is responsive to the Tre6P–Suc nexus, which allows for the release of SnRK1-mediated carbon accumulation when sucrose levels increase beyond an optimum, redirecting the flow of carbon towards glycolysis, the TCA cycle, and protein and lipid synthesis. Thick black arrows denote a positive effect of SnRK1 on the indicated metabolic process. Asterisks indicate components affected by SnRK1 at the level of enzyme activity and transcript accumulation. AGPase, ADPGlc pyrophosphorylase; CCT, CTP:phosphocholine cytidyltransferase; cyt-FBPase, cytosolic fructose-1,6-bisphosphatase; DGAT1, diacylglycerol transferase1; F2KP, fructose-6-phosphate 2-kinase/fructose-2,6-bisphosphatase; Fru-2,6bP, fructose-2,6-bisphosphate; INV, invertase; np-Ga3PDHase, non-phosphorylating glyceraldehyde-3-phosphate dehydrogenase; NR, nitrate reductase; O2, Opaque2; PFP, pyrophosphate-dependent phosphofructokinase; SPS, sucrose phosphate synthase; SS, starch synthase; SUSY, sucrose synthase; Tre6P, trehalose 6-phosphate; WRI1, Wrinkled1.

In source leaves and in developing sink organs (Fig. 1), SnRK1 appears to fine-tune metabolism to promote Suc and starch synthesis at the expense of glycolysis, organic acids, amino acids, and lipids. By inducing the conversion of Suc into starch, SnRK1 would further facilitate the import of Suc from the phloem and hence promote sink strength. Feedback regulation from the Suc–Tre6P system in turn would down-regulate basal SnRK1 activity when Suc levels rise, redirecting the flux of carbon from Suc into glycolysis and the TCA cycle, nitrogen assimilation, and lipid synthesis. One core function of SnRK1 kinases would therefore be the maintenance of Suc homeostasis, playing an equivalent role to that of mammalian AMPK in the control of blood Glc levels (Long and Zierath, 2006). Maintaining stable Suc levels may be important to ensure adequate growth of the sinks while avoiding detrimental effects derived from excessive sugar accumulation particularly in the source organs (Paul and Foyer, 2001). Such a function would be central for the coordination of source and sink activities and is compatible with the metabolic alterations reported in source and sink organs of plants manipulated for SnRK1. This would further enable a coordination of carbon and nitrogen metabolism, as also postulated for Tre6P (Figueroa *et al.*, 2016). Homeostatic control of Suc levels may also be important for establishing a balance between growth and stress responses, ensuring that growth is not freely released but is rather accompanied by the ability to rapidly respond to unfavourable conditions. In response to stress, this homeostatic control system would be transiently overridden by severe SnRK1 activation, shifting the balance towards stress responses, and putting in place mechanisms to cope with an energy crisis.

**Fig. 2.**
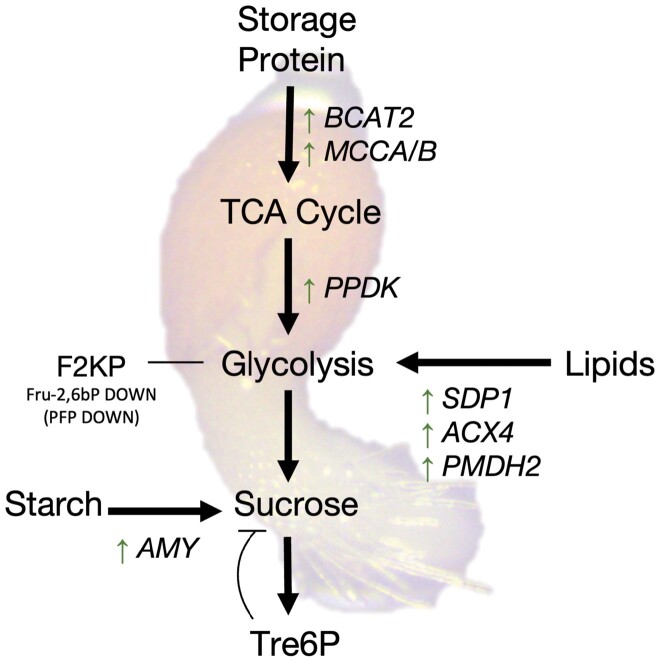
Model of SnRK1-mediated metabolic adaptations in germinating seedlings. SnRK1 acts by regulating metabolic enzymes and transcription factors (see text for details), leading to an increase (green arrows) or decrease (red arrows) in their activities. Lack of green/red arrows in F2KP indicates that the outcome of SnRK1-mediated regulation on enzyme activity is unclear. During germination and early seedling development, SnRK1 promotes the remobilization of reserves (starch, storage protein, or lipids) to fuel normal metabolism and growth, potentially also contributing to the maintenance of optimal sucrose levels. Thick black arrows denote a positive effect of SnRK1 on the indicated metabolic process. Italic, transcripts affected by SnRK1 activity. ACX4, acyl-CoA oxidase4; AMY, amylase; BCAT2, branched chain amino acid transaminase2; F2KP, fructose-6-phosphate 2-kinase/fructose-2,6-bisphosphatase; Fru-2,6bP, fructose-2,6-bisphosphate; MCCA/B, methylcrotonyl-CoA carboxylase subunit A/B; PFP, pyrophosphate-dependent phosphofructokinase; PMDH2, peroxisomal malate dehydrogenase2; PPDK, pyruvate orthophosphate dikinase; SDP1, sugar dependent1; Tre6P, trehalose 6-phosphate.

In other stages of development where remobilization of resources is required (Fig. 2), such as germination and early seedling development, SnRK1 promotes starch, lipid, and amino acid degradation to provide energy for seedling growth. Such functions would be similar to those performed during the stress response and may be amplified if the germinating seedling encounters unfavourable conditions (Henninger *et al.*, 2022). Ultimately, one major function also in this case would be to supply and maintain Suc at optimal levels for growth.

It is important to note that our conclusions rely on the steady-state characterization of metabolic changes when SnRK1 is constitutively manipulated, making it impossible to pinpoint what the primary effects are. Future research should therefore employ more dynamic analyses of metabolism (e.g. flux analyses) to enable a precise determination of the steps that are affected by SnRK1. On the other hand, SnRK1 activity is intimately linked to that of the TOR kinase under both favourable and stressful conditions (Jamsheer K *et al*., 2019, 2022; Rodriguez *et al.*, 2019), making it hard to distinguish whether the observed metabolic changes are directly driven by SnRK1 or indirectly via TOR. Furthermore, this involvement in the regulation of growth and development calls for caution when interpreting results derived from the acute/strong overexpression or silencing of SnRK1, as the observed metabolic changes may be indirect and caused by decreased growth. Future studies should therefore consider the possible involvement of TOR in the measured outputs as well as the specificity, timing, and strength of SnRK1 manipulation, as all of these factors could feed back into metabolism in unpredictable ways. Constitutive gain- and loss-of-function mutants will still be relevant in further understanding how SnRK1 is important for coordinating carbon and nitrogen metabolism between different tissues and at different developmental stages, but future studies will inevitably have to rely on more precise manipulation of these components in specific tissues or developmental stages to minimize pleiotropic effects.

Metabolic analyses should also be increasingly complemented with more molecular studies to investigate and further identify metabolic enzymes that interact with and are phosphorylated by SnRK1, and to further integrate these findings into the different metabolic pathways. In this context, it would also be important to address how SnRK1 could access these enzymes *in vivo,* given that many of them localize to plastids and other organelles.

## Supplementary data

The following supplementary data are available at *JXB* online.

Table S1. Overview of the metabolic consequences of SnRK1α genetic manipulation.

## Supplementary Material

erac261_suppl_Supplementary_Table_S1
